# A clinical evaluation committee assessment of recombinant human tissue factor pathway inhibitor (tifacogin) in patients with severe community-acquired pneumonia

**DOI:** 10.1186/cc7747

**Published:** 2009-03-15

**Authors:** Pierre-François Laterre, Steven M Opal, Edward Abraham, Steven P LaRosa, Abla A Creasey, Fang Xie, Lona Poole, Richard G Wunderink

**Affiliations:** 1 St Luc University Hospital, Université Catholique de Louvain, Avenue Hippocrate 10, 1200 Brussels, Belgium; 2 Division of Infectious Diseases, Rhode Island Hospital, POB Suite #330, 593 Eddy Street, Providence, RI 02903, USA; 3 Department of Medicine, University of Alabama at Birmingham School of Medicine, 420 Boshell Building, 1808 7th Avenue South, Birmingham, AL 35294, USA; 4 Alza Corporation, Johnson & Johnson, 1900 Charleston Road, Mountain View, CA 94042, USA; 5 Novartis, 4560 Horton Street, Emeryville, CA 94608, USA; 6 Division of Pulmonary and Critical Care Medicine, Northwestern University Feinberg School of Medicine, 240 E. Huron Street, McGaw M300, Chicago, IL 60611, USA

## Abstract

**Introduction:**

The purpose of this analysis was to determine the potential efficacy of recombinant human tissue factor pathway inhibitor (tifacogin) in a subpopulation of patients with community-acquired pneumonia (CAP) from a phase III study of severe sepsis.

**Methods:**

A retrospective review of patients with suspected pneumonia was conducted by an independent clinical evaluation committee (CEC) blinded to treatment assignment. The CEC reanalyzed data from patients enrolled in an international multicenter clinical trial of sepsis who had a diagnosis of pneumonia as the probable source of sepsis. The primary efficacy measure was all-cause 28-day mortality.

**Results:**

Of 847 patients identified on case report forms with a clinical diagnosis of pneumonia, 780 (92%) were confirmed by the CEC to have pneumonia. Of confirmed pneumonia cases, 496 (63.6%) met the definition for CAP. In the CEC CAP population, the mortality rates of the tifacogin and placebo groups were 70/251 (27.9%) and 80/245 (32.7%), respectively. The strongest signals were seen in patients with CAP not receiving concomitant heparin, having microbiologically confirmed infection, or having the combination of documented infection and no heparin. The reduction in mortality in this narrowly defined subgroup when treated with tifacogin compared with placebo was statistically significant (17/58 [29.3%] with tifacogin and 28/54 [51.9%] with placebo; unadjusted *P *value of less than 0.02).

**Conclusions:**

Tifacogin administration did not significantly reduce mortality in any severe CAP patient. Exploratory analyses showed an improved survival in patients who did not receive concomitant heparin with microbiologically confirmed infections. These data support the rationale of an ongoing phase III study exploring the potential benefit of tifacogin in severe CAP.

**Trial Registration:**

ClinicalTrials.gov identifier NCT00084071.

## Introduction

Sepsis is a systemic response to infection associated with significant mortality and substantial direct patient care costs [1]. Community-acquired pneumonia (CAP) is the most common cause of sepsis [2-5]. CAP mortality rates are significant and have not changed significantly over several decades despite the availability of improved broad-spectrum antibiotics [6]. While successful outcome from severe CAP requires adequate treatment of the infection, antimicrobial agents alone have only limited capacity to reduce the mortality rate associated with severe CAP and adjunctive measures are required to treat organ dysfunction such as respiratory failure [6].

Likely contributors to organ dysfunction and death are intravascular and intrapulmonary generation of thrombin and deposition of fibrin due to break down in hemostatic regulation. Increased cell surface expression of tissue factor (TF) in severe CAP induces thrombin generation and fibrin formation [7,8]. TF expression in the lungs of pneumonia patients leads to a proinflammatory and procoagulant environment as well as to decreased fibrinolysis [9].

TF pathway inhibitor (TFPI) regulates coagulation initiated by TF. Expression of TF and TFPI is imbalanced in acute lung injury [10]. Administration of recombinant TFPI or factor VIIa antagonists reduces lung injury and systemic cytokine responses in infection models [11-14]. Therefore, TF inhibition may have beneficial effects in disease states such as acute lung injury or pneumonia in which coagulation and inflammation play prominent roles [9].

Safety and efficacy of tifacogin, a recombinant form of human TFPI, were assessed in a phase III study (TFP007 OPTIMIST [Optimized Phase III Tifacogin in Multicenter International Sepsis Trial]) in patients with severe sepsis [15]. Although efficacy of the primary endpoint of 28-day all-cause mortality was not shown, treatment benefit in a subset of patients with pneumonia with microbiological documentation and not receiving heparin within 24 hours prior to and/or during study drug infusion was observed in *post hoc *analysis. However, these analyses were based on case report forms (CRFs) in which investigators were allowed to list multiple sites of infection and any positive cultures. Not all positive cultures grew pathogens, and the organisms grown were not necessarily consistent with the suspected infection site.

Concern regarding the accuracy of subgroup classification in TFP007 prompted the creation of a clinical evaluation committee (CEC) to validate the CRF-based analyses. CECs have previously been engaged to evaluate negative trials of adjuvant agents in critical illness in order to determine a target population for further study [16,17]. The CEC was specifically charged with determining (a) the validity of the pneumonia diagnosis, (b) whether the pneumonia was CAP, hospital-acquired pneumonia (HAP), or other diagnoses, and (c) the level of evidence of a microbiological etiology of CAP.

## Materials and methods

A detailed description of the study was previously published [15]. The OPTIMIST study was approved by the ethics committee of each individual participating center, and written consent was obtained from each patient or next of kin. The CEC retrospective study was approved by the ethics committee of St Luc University Hospital (Brussels, Belgium). Initial analyses of the TFP007 patient subgroup with pneumonia used a programmatic definition of CAP that allowed a maximum of 2 days of hospitalization prior to the start of study drug for the pneumonia to be classified as CAP. Patients hospitalized longer than 2 days were classified as having HAP.

The CEC consisted of critical care, pulmonary disease, and infectious disease specialists who remained blinded to treatment throughout the evaluation. A charter incorporating a predetermined set of clinical and microbiological classification rules was used to ensure uniformity of this retrospective assessment [18]. Criteria to be classified as CAP included all five of the following: (a) the clinical and radiographic evidence was consistent with pneumonia, (b) microbiology (when provided) was consistent with a CAP pathogen, (c) the primary reason for hospital admission was pneumonia, (d) there was no evidence of aspiration or major immunocompromised state, and (e) the patient was not a known nursing home resident or transfer from another institution. Chest x-ray protocol provided by a radiologist at each investigator site was used to define evidence of CAP. In the CEC analysis, the CAP time window was expanded to 4 days between hospital admission and start of study drug infusion for cases with signs and symptoms of CAP on admission. This interval was chosen based on the time windows used for patient enrollment [19] and to include CAP patients who deteriorated after admission [20,21].

TFP007 investigators classified 847 patients as having CAP on the CRFs. Cases in which pneumonia was not listed by the investigator as a potential site of infection were not reviewed. The CEC reviewed all available information on CRFs from the locked TFP007 database for those subjects. Each case was independently reviewed by one member, and then the CEC met to reach a consensus on all problematic cases. No adjudication of any outcome data was performed.

The CEC assessment forms were tabulated, and the tifacogin arm was compared with placebo for all CEC-confirmed CAP cases. Additional analyses were carried out for microbiological evidence (based on culture only), heparin use, serious bleeding events and contributing causes, and the Acute Physiology and Chronic Health Evaluation II (APACHE II) score quartiles [4,22]. The results of the CEC evaluation were compared with those of the original programmatic classification.

Elevated procalcitonin (PCT) (>0.5 ng/mL) levels are associated with a bacterial etiology in patients presenting with suspected pulmonary infection [23]. PCT levels were measured in plasma specimens prospectively collected (Brahms AG, Hennigsdorf, Germany). The CEC classification was performed without knowledge of PCT values. PCT levels were evaluated in the CEC-designated CAP population. Chi-square tests were used to compare treatment groups for dichotomous variables. All *P *values are unadjusted for multiple testing. Logistic regression models were also used to adjust for baseline APACHE II score, PCT level, shock, and use of ventilator support.

## Results

### Confirmation of pneumonia and community-acquired pneumonia diagnosis

In its review of 847 patients identified on CRFs with a diagnosis of pneumonia, the CEC concurred that pneumonia was the cause of sepsis in 780 cases (92%). One patient could not be evaluated. Of the 780 confirmed pneumonia cases, 496 were classified by the CEC as CAP (251 in the tifacogin group and 245 in the placebo group) and 259 were classified as HAP (132 in the tifacogin group and 127 in the placebo group). Because of a major immunocompromised state, aspiration, or radiation pneumonitis, 25 patients did not meet the standard definition of CAP and therefore were excluded. The CRF-defined pneumonia subgroup identified pneumonia patients misclassified as having CAP when they actually had HAP and vice versa.

Demographics of the 496 CEC CAP patients are presented in Table 1. Sixty-eight percent received heparin and 65% had a microbiologically confirmed infection. Baseline characteristics in the placebo and tifacogin groups were similar. For both documented CAP and no-heparin-use subgroups, baseline APACHE II scores and presence of shock did not differ between TFPI and placebo.

**Table 1:** Baseline demographics for clinical evaluation committee community-acquired pneumonia patients

	Tifacogin n = 251	Placebo n = 245	Selected *P *value
Age in years, mean ± standard deviation	60.5 ± 15.8	60.2 ± 15.1	
Gender, number (percentage)			
Female	91 (36)	100 (41)	
Male	160 (64)	145 (59)	
Ethnicity, number (percentage)			
Caucasian	207 (82)	199 (81)	
Black	26 (10)	20 (8)	
Hispanic	9 (4)	12 (5)	
Asian	5 (2)	7 (3)	
Other	4 (2)	7 (3)	
Baseline APACHE II score			
Mean ± standard deviation	25.6 ± 7.0	25.2 ± 6.7	
Baseline interleukin-6	434.6	478.9	
Geometric mean (95% CI)	(329.2, 573.6)	(355.1, 645.9)	
Baseline procalcitonin			
Geometric mean (95% CI)	8.89 (7.15, 11.05)	8.09 (6.35, 10.31)	
<2 ng/mL, number (percentage)	50 (20)	60 (25)	
Shock, number (percentage)	163 (65)	176 (72)	0.10
Ventilatory support, number (percentage)	193 (77)	200 (82)	0.19
Number of organ dysfunctions, number (percentage)			
Two or less	87 (35)	72 (29)	0.21
Three or more	164 (65)	173 (71)	
Heparin use, number (percentage)	172 (69)	167 (68)	
Organism identified, number (percentage)	170 (68)	154 (63)	

APACHE II, Acute Physiology and Chronic Health Evaluation II; CI, confidence interval.

The spectrum of etiologic microorganisms is presented in Table 2. *Streptococcus pneumoniae *was the most common pathogenic organism. Twelve cases had only sputum Gram stain evidence of pneumonia. Baseline PCT levels above 2 ng/mL were present in the majority (78%) of CEC CAP patients. Only 60/245 (25%) of placebo and 50/251 (20%) tifacogin-treated patients had PCT levels of less than 2 ng/mL.

**Table 2:** Clinical evaluation committee classification of causative microorganisms in community-acquired pneumonia patients

	Tifacogin n = 251		Placebo N = 245	
Organism identified	Number (percentage)	Mortality, number (percentage)	Number (percentage)	Mortality, number (percentage)
*Streptococcus pneumoniae*	69 (27.5)	14 (20.3)	70 (28.6)	19 (27.1)
*Staphylococcus aureus*	33 (13.1)	14 (42.4)	19 (7.8)	12 (63.2)
*Haemophilus influenzae*	26 (10.4)	5 (19.2)	10 (4.1)	5 (50)
Other respiratory organisms	24 (9.6)	9 (37.5)	23 (9.4)	6 (26.1)
Enteric Gram-negative	20 (8.0)	6 (30)	16 (6.5)	9 (56.3)
Gram stain only	9 (3.6)	3 (33.3)	9 (3.7)	4 (44.4)
Legionella species	6 (2.4)	1 (16.7)	10 (4.1)	2 (20)
*Pseudomonas aeruginosa*	6 (2.4)	1 (16.7)	12 (4.9)	5 (41.7)
Respiratory viruses	2 (0.8)	1 (50)	0	0
*Chlamydophila pneumoniae*	0	0	2 (0.8)	0
No organism identified	81 (32.3)		91 (37.1)	

### Effect of tifacogin in clinical evaluation committee community-acquired pneumonia cohort

**Figure 1:**
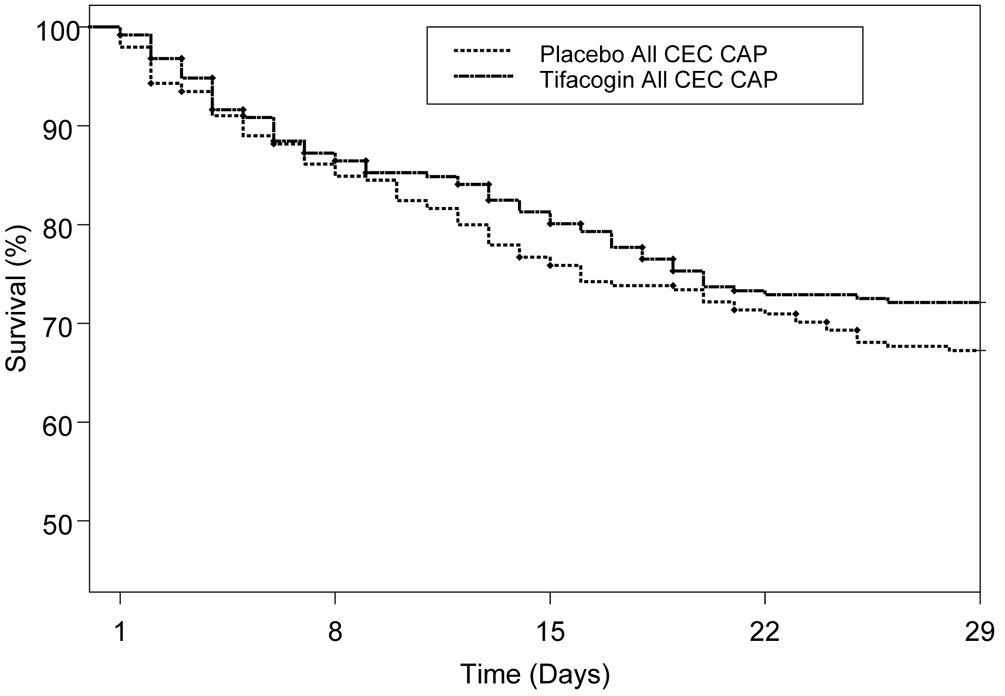
Kaplan-Meier survival analysis for all clinical evaluation committee (CEC) community-acquired pneumonia (CAP) patients. *P *value = 0.25.

**Figure 2:**
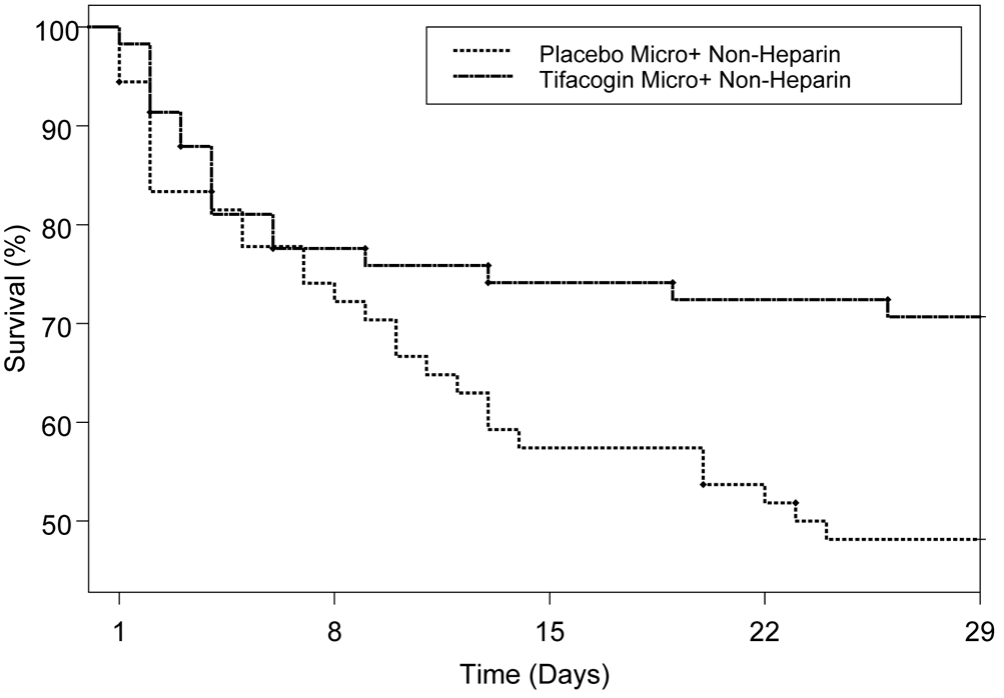
Kaplan-Meier survival analysis for clinical evaluation committee community-acquired pneumonia patients in the non-heparin cohort with microorganism identified. *P *value = 0.02.

Kaplan-Meier plots of 28-day cumulative survival data for all CEC CAP patients (Figure 1) and for CEC CAP subjects who did not receive heparin and who also had microbiological evidence of an infectious etiology for their pneumonia (Figure 2) are shown. The CEC CAP patients treated with tifacogin had lower 28-day all-cause mortality compared with the placebo group (27.9% versus 32.7%; *P *= 0.25, Pearson chi-square test; *P *= 0.22, logistic regression model). The largest difference in 28-day mortality (Table 3 and Figure 2) occurred in the subgroup of patients with microbiological evidence of infection in the no-heparin CAP cohort (29.3%, tifacogin; 51.9%, placebo; *P *= 0.02, Pearson chi-square test; *P *= 0.02, logistic regression model).

**Table 3:** Mortality (28-day) in tifacogin and placebo groups for all patients and by microbiology status and heparin use

	Tifacogin			Placebo			*P *value
	All	Mortality		All	Mortality		
	Number	Number	Percentage	Number	Number	Percentage	
All community-acquired pneumonia patients	251	70	27.9	245	80	32.7	0.25
Microbiology status							
Organism identified	170	46	27.1	154	55	35.7	0.09
Organism not identified	81	24	29.6	91	25	27.5	0.75
Procalcitonin level							
<2	50	15	30.0	60	18	30.0	1.00
≥ 2	200	55	27.5	183	62	33.9	0.18
Heparin use							
No Heparin	79	23	29.1	78	33	42.3	0.08
Heparin	172	47	27.3	167	47	28.1	0.87
Microbiology status and heparin use							
No heparin/Organism identified	58	17	29.3	54	28	51.9	0.02
No heparin/Organism not identified	21	6	28.6	24	5	20.8	0.54
Shock							
Yes	163	50	30.7	176	64	36.4	0.27
No	88	20	22.7	69	16	23.2	0.95
Ventilatory support							
Yes	193	60	31.1	200	75	37.5	0.18
No	58	10	17.2	45	5	11.1	0.38
Number of organ dysfunctions							
Two or less	87	17	19.5	72	13	18.1	0.81
Three or more	164	53	32.3	173	67	38.7	0.22

### Effect of pathogen class and causative microorganism on mortality

**Figure 3:**
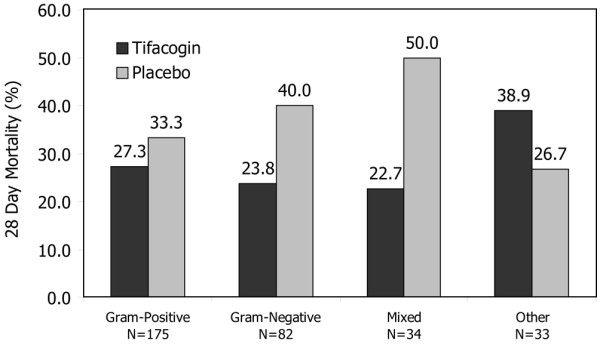
Mortality by bacterial Gram stain morphology in clinical evaluation committee community-acquired pneumonia patients.

The observed mortality of the tifacogin-treated group was lower than that of the placebo group for all pathogen classes (Gram-positive, Gram-negative, mixed, and other) (Figure 3) and when analyzed by individual pathogen, except unusual respiratory pathogens and respiratory viruses (Table 2). For *S. pneumoniae*, the observed 28-day mortality in tifacogin-treated subjects was 20.3% versus 27.1% in the placebo arm. When analyzed by PCT levels of less than 2 ng/mL and of greater than or equal to 2 ng/mL, the observed mortality in the tifacogin-treated cohort was improved in subjects with higher PCT levels (Table 3).

### Tifacogin treatment effect and APACHE II score

**Figure 4:**
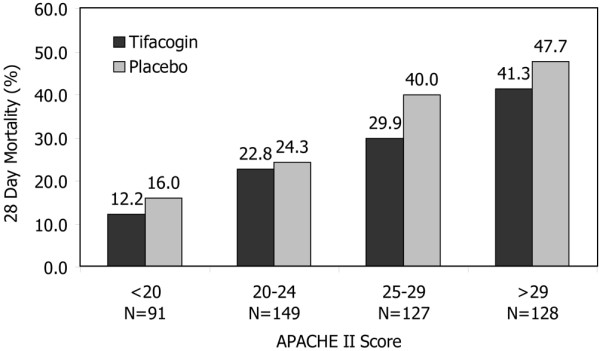
Mortality by Acute Physiology and Chronic Health Evaluation II (APACHE II) score quartiles in clinical evaluation committee community-acquired pneumonia patients treated with tifacogin or placebo. The quartiles were determined by PROWESS (Recombinant Human Activated Protein C Worldwide Evaluation in Severe Sepsis) trial results.

Based on PROWESS (Recombinant Human Activated Protein C Worldwide Evaluation in Severe Sepsis) [4,22], CEC CAP patients were segregated into quartiles of APACHE II scores of less than 20 (n = 91), 20 to 24 (n = 149), 25 to 29 (n = 127), and greater than 29 (n = 128) (Figure 4). In both tifacogin and placebo groups, mortality increased with higher APACHE II scores. Mortality for patients receiving tifacogin was lower than mortality in the placebo group in all four APACHE II score quartiles.

### Safety

The overall incidence of all adverse events was similar in the tifacogin group (93%) and the placebo group (91%). Serious adverse event rates, likewise, were similar (41% versus 52%, respectively). Since tifacogin is an anticoagulant, the incidence of events involving bleeding was scrutinized. Tifacogin-treated CAP patients had higher rates of bleeding events (23% versus 18%) and serious bleeding events (6% versus 2%) compared with placebo CAP patients. The most common sites of bleeding were the gastrointestinal and respiratory tracts in the tifacogin group and the gastrointestinal tract and skin (ecchymoses) in the placebo group. Higher rates of bleeding events occurred in subgroups receiving concomitant heparin (Tables 4 and 5) than in those not treated with heparin.

**Table 4:** Incidence of serious bleeding adverse events in patients with and without concomitant heparin use

MedDRA system organ class	Number (percentage) of subjects^a^			
	Heparin cohort		Non-heparin cohort	
	Tifacogin	Placebo	0.025 TFPI	Placebo
	(n = 172)	(n = 167)	(n = 79)	(n = 78)
Any serious adverse event	11 (6%)	4 (2%)	3 (4%)	2 (3%)
Gastrointestinal disorders	1 (1%)	2 (1%)	2 (3%)	2 (3%)
General disorders and administration site condition	1 (1%)	1 (1%)	0	0
Injury and poisoning	0	1 (1%)	0	0
Nervous system disorders	3 (2%)	0	0	0
Respiratory, thoracic, and mediastinal disorders	1 (1%)	0	0	0
Surgical and medical procedures	2 (1%)	0	0	0
Vascular disorders	3 (2%)	0	1 (1%)	0

^a^Number and percentage of subjects with one or more events that map to each MedDRA system organ class. Hence, MedDRA system organ class counts may not equate with overall counts. MedDRA, Medical Dictionary for Regulatory Activities; TFPI, tissue factor pathway inhibitor.

**Table 5:** Incidence of central nervous system bleeding events in placebo- and tifacogin-treated patients with and without concomitant heparin use

MedDRA system organ class	Number (percentage) of subjects^a^			
	Heparin cohort		Non-heparin cohort	
	Tifacogin	Placebo	0.025 TFPI	Placebo
	(n = 172)	(n = 167)	(n = 79)	(n = 78)
Any serious adverse event	5 (3%)	0	1 (1%)	0
Nervous system disorders	3 (2%)	0	0	0
Vascular disorders	2 (1%)	0	1 (1%)	0

^a^Number and percentage of subjects with one or more events that map to each MedDRA system organ class. Hence, MedDRA system organ class counts may not equate with overall counts. MedDRA, Medical Dictionary for Regulatory Activities; TFPI, tissue factor pathway inhibitor.

## Discussion

Retrospective subgroup analyses may identify potential target populations for future trials. The OPTIMIST trial [15] showed no improvement in mortality with tifacogin compared with placebo. Overall, the 28-day survival in patients with CAP treated with tifacogin was higher compared with placebo but the difference did not reach statistical significance. However, subgroup analysis of this study suggested that patients not receiving heparin and/or with microbiological evidence of pneumonia appeared to benefit from tifacogin. Using blinded and stringent evaluations, the CEC strengthened the database used for reanalysis, demonstrating an important role for CECs in retrospective review. The CEC analysis corroborated the initial analysis by showing a reduction in mortality in the tifacogin-treated CAP subgroup, not receiving heparin, with microbiologically confirmed infection, or when these two conditions were present.

Benefit of an agent affecting the coagulation pathway in a population with pneumonia versus other sources of infection has biological plausibility. In animal models of acute bronchopneumonia, activation of coagulation can be readily demonstrated [24,25]. Bronchoalveolar lavage specimens from patients with acute lung injury also indicate activation of coagulation [26]. Recombinant human activated protein C (aPC), an anticoagulant approved for the treatment of severe sepsis, had its greatest benefit in the population with severe CAP in a similar CEC analysis [19].

PCT has been shown to be consistently elevated in bacterial infections [23,27]. The beneficial effect of tifacogin in patients with levels above 2 ng/mL reinforces the need for the phase III confirmatory study to emphasize documented bacterial CAP. Both microbiological data and the PCT levels suggest that tifacogin may have a disproportionate beneficial effect in microbiologically confirmed cases of CAP.

The finding of a beneficial effect of tifacogin in patients with microbiologically confirmed infection has several potential explanations. Opal and colleagues [28] demonstrated that patients with severe sepsis with a microbiologically confirmed infection had greater perturbations of their coagulation and inflammatory parameters compared with patients with culture-negative severe sepsis. The ability to recover an organism may indicate a patient with greater activation of the coagulation system, a more pronounced proinflammatory stimulus, or both. Recombinant human TFPI binds to lipopolysaccharides (LPSs) and blocks LPS interaction with LPS-binding protein [29]. Endotoxemia may occur in both Gram-positive and Gram-negative cases of pneumonia [30]. This finding raises the possibility that tifacogin exerts a beneficial effect via immune signaling activities. Finally, tifacogin could potentially play a role in aiding bacterial clearance, which would explain this differential benefit in culture-positive cases [31]. Therefore, three potential mechanisms of action whereby tifacogin may benefit patients with severe CAP are (a) coagulation regulation, (b) immune modulation, and (c) bacterial clearance. The clinical relevance of this hypothesis remains unknown.

In contrast to results in the no-heparin cohort, no benefit of tifacogin was found in CAP patients who received heparin. This result can possibly be explained by potential interactions of tifacogin and heparin. TFPI is most active when expressed on the surface of the cell [32]. Heparin initiates intracellular signaling that results in the transfer of endothelial cell surface-bound TFPI to intracellular storage vesicles, decreasing activity. Heparin could also result in TFPI release into the bloodstream, where it is eventually degraded and is no longer active. In addition, the heparin-binding site on TFPI overlaps the LPS-binding site in the third Kunitz region and carboxyl terminus and competes with TFPI LPS binding [30]. Such an effect could interfere with tifacogin biological activity, suggesting the possibility of a true drug-drug interaction to explain the neutralization of beneficial effect of tifacogin by heparin.

An apparent mortality benefit of heparin use in the placebo group has been noted in several sepsis trials using anticoagulant therapies. However, patients were not randomly assigned to heparin or no-heparin treatment; they were randomly assigned to the study drug only. Investigators used heparin at their discretion and it is reasonable to assume that heparin use would be selected for patients who were less critically ill and less likely to have major coagulopathies. Patients who died early in the course of their illness after random assignment did not have the opportunity to receive heparin. While the benefit of heparin is likely due to the unequal allocation and selection bias [17], a beneficial effect of heparin alone cannot be excluded. A randomized controlled trial of unfractionated heparin for sepsis is currently under way (ClinicalTrials.gov identifier NCT00100308). However, because of both potential confounding and the possible drug-drug interaction, the phase III confirmation study will require exclusion of heparin therapy during the time of active treatment. TFPI has not been demonstrated to be efficacious for the prevention of deep venous thrombosis in critically ill patients. Mechanical compression devices, an acceptable alternative for critically ill patients at increased risk of bleeding (American College of Chest Physicians guidelines), would therefore be required for both treated and placebo groups.

An additional finding in the CEC CAP subgroup is the apparent absence of a disease severity interaction. Though not reaching statistical significance, the mortality rates in the tifacogin-treated arm were consistently lower than those in the placebo arm in all four APACHE II score quartiles. This finding is unlike results of other clinical trials involving anti-inflammatory compounds and aPC [33].

Incidence rates of adverse events and events associated with bleeding in CAP patients receiving tifacogin were similar to those in the original TFP007 patient population [15]. Bleeding risk increased in CAP patients receiving both heparin and tifacogin, further emphasizing that tifacogin should not be coadministrated with heparin. Most patients who experienced serious bleeding events had pre-existing conditions that put them at increased risk for hemorrhagic complications.

CEC analyses of large phase III databases have recognized limitations. These evaluations are retrospective in nature and are based on progressively smaller subgroup sizes, leading to an increasing potential for error. Retrospective analyses of CAP patients' data include an additional hazard: they lack assessment of adequacy of antimicrobial therapy. As is typical of retrospective subgroup analyses, this analysis of a small subgroup of severe CAP patients is solely hypothesis-generating. A subsequent study to test the hypothesis developed by subgroup analysis is more likely to succeed if underlying biological principles support the use of the molecule in that defined population. While the statistical tests are not corrected for the number of subgroups examined, these data and supportive evidence from the literature strengthen the hypothesis that the best target for tifacogin is a population with severe CAP in the absence of concomitant heparin use.

## Conclusions

From this retrospective review of patients with severe CAP evaluating the role of tifacogin administration, exploratory analyses showed an improved survival in patients with documented infections who did not receive concomitant heparin. These data support the rationale of the phase III double-blind randomized controlled study exploring the potential benefit of tifacogin in patients with severe CAP admitted to the intensive care unit.

## Key messages

• From this retrospective analysis, tissue factor pathway inhibitor seems to improve outcome in severe documented community-acquired pneumonia.

• Concomitant heparin use seems to suppress this observed benefit.

• A prospective randomized controlled study is warranted to confirm this hypothesis.

## Abbreviations

APACHE II: Acute Physiology and Chronic Health Evaluation II; aPC: activated protein C; CAP: community-acquired pneumonia; CEC: clinical evaluation committee; CRF: case report form; HAP: hospital-acquired pneumonia; LPS: lipopolysaccharide; OPTIMIST: Optimized Phase III Tifacogin in Multicenter International Sepsis Trial; PCT: procalcitonin; TF: tissue factor; TFPI: tissue factor pathway inhibitor.

## Competing interests

P-FL has been a consultant for, has participated in advisory boards to, and has received lecture fees from Eli Lilly and Company (Indianapolis, IN, USA), Novartis (formerly Chiron, Emeryville, CA, USA), and GlaxoSmithKline (Uxbridge, Middlesex, UK). SMO is funded by Wyeth Research (Madison, NJ, USA) for preclinical research. He serves as an investigator for the Ocean State Clinical Coordinating Center (Providence, RI, USA), which is funded by Novartis (East Hanover, NJ, USA) and Eisai Medical Research (Woodcliff Lake, NJ, USA) for the conduct of clinical trials for the treatment of sepsis. EA was one of the principal investigators for the TFP007 study, and his institution received a contract from Chiron for patient enrollment. Since 2004, he has not received any consulting income or any other funds from Chiron/Novartis or any entity with interest in the subject of this manuscript. SPL has received consulting fees from Eisai Medical Research and Chiron/Novartis for serving on the CEC and has received investigator grants from these companies for serving on the Ocean State Clinical Coordinating Center. AAC, FX, and LP are current or former Chiron/Novartis employees. RGW has been paid on an hourly basis for work on the CEC and has also received an investigator-initiated grant from Chiron/Novartis.

## Authors' contributions

P-FL, SMO, SPL, and RGW participated in the study design, in the acquisition and interpretation of the data, and in the drafting of the manuscript. EA, AAC, FX, and LP participated in the interpretation of the data and in the drafting of the manuscript. All authors read and approved the final manuscript.

## Authors' information

This work was performed at Novartis (formerly Chiron, Emeryville, CA, USA).
